# Implementing a Scoring Function Based on Interaction Fingerprint for Autogrow4: Protein Kinase CK1δ as a Case Study

**DOI:** 10.3389/fmolb.2022.909499

**Published:** 2022-07-07

**Authors:** Matteo Pavan, Silvia Menin, Davide Bassani, Mattia Sturlese, Stefano Moro

**Affiliations:** Molecular Modeling Section (MMS), Department of Pharmaceutical and Pharmacological Sciences, University of Padova, Padova, Italy

**Keywords:** fragment-based drug discovery, protein kinase CK1δ, neurodegenerative diseases, Autogrow, interaction fingerprint, *de novo* drug design, fragment growing, lead optimization

## Abstract

In the last 20 years, fragment-based drug discovery (FBDD) has become a popular and consolidated approach within the drug discovery pipeline, due to its ability to bring several drug candidates to clinical trials, some of them even being approved and introduced to the market. A class of targets that have proven to be particularly suitable for this method is represented by kinases, as demonstrated by the approval of BRAF inhibitor vemurafenib. Within this wide and diverse set of proteins, protein kinase CK1δ is a particularly interesting target for the treatment of several widespread neurodegenerative diseases, such as Alzheimer’s disease, Parkinson’s disease, and amyotrophic lateral sclerosis. Computational methodologies, such as molecular docking, are already routinely and successfully applied in FBDD campaigns alongside experimental techniques, both in the hit-discovery and in the hit-optimization stage. Concerning this, the open-source software Autogrow, developed by the Durrant lab, is a semi-automated computational protocol that exploits a combination between a genetic algorithm and a molecular docking software for *de novo* drug design and lead optimization. In the current work, we present and discuss a modified version of the Autogrow code that implements a custom scoring function based on the similarity between the interaction fingerprint of investigated compounds and a crystal reference. To validate its performance, we performed both a *de novo* and a lead-optimization run (as described in the original publication), evaluating the ability of our fingerprint-based protocol to generate compounds similar to known CK1δ inhibitors based on both the predicted binding mode and the electrostatic and shape similarity in comparison with the standard Autogrow protocol.

## GRAPHICAL ABSTRACT

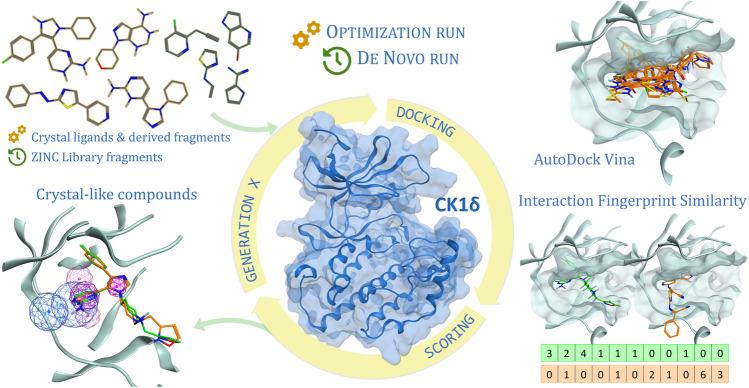

## 1 Introduction

Protein kinase CK1δ is a Ser/Thr protein kinase belonging to the casein kinase 1 family. In mammals, seven distinct genes encoding for casein kinase proteins are present, each producing a different isoform (α, β, γ1, γ2, γ3, δ, and ε) (Knippschild et al., 2005). CK1 family proteins use exclusively ATP as a phosphate source for their kinase activity, which is carried out by the protein in its monomeric form. Each isoform is constitutionally active and does not require the presence of a cofactor to exert its activity (Knippschild et al., 2014).

From a biological function point of view, the members of this family have been historically related to different physiological mechanisms, such as cell replication (Xu et al., 2019), DNA repair (Behrend et al., 2000), and circadian rhythm (Lee et al., 2009).

From a structural perspective, the members of the CK1 family are characterized by the typical bilobed structure of the globular Ser-Thr kinase proteins, with the N-term lobe consisting mainly of β-sheets, and a larger C-term lobe, constituted primarily of α-helices. The two domains are connected by a protein region named the “hinge region,” which forms a highly conserved pocket for ATP binding (Knippschild et al., 2014).

As for other members of the CK1 family, CK1δ recognizes the canonical phospho-primed structural motif *pSer/pThr-X*_*1-2*_ *-Ser/Thr*, where X stands for any amino acid and pSer/pThr represents the phospho-primed residue (Meggio et al., 1991). The CK1 kinases are also able to recognize non-phosphorylated sequences, as far as they contain strongly acidic residues (Asp or Glu) that can make up for the absence of the phosphorylated residue (Xu et al., 2019). The structural motif that can be recognized by the CK1 proteins is widespread in many cellular proteins and, because of this, over 140 substrates have been reported both *in vitro* and *in vivo* (Knippschild et al., 2014), underlining the pleiotropic character of this protein family. Due to the great variability of its substrates, CK1δ is involved in many cellular pathways, among which the main ones are the Wnt-pathway, the Hippo pathway, the p53 regulation pathway, and the Hedgehog pathway (Xu et al., 2019).

The endogenous regulation of CK1δ, on the other hand, can be carried out through various mechanisms, including autophosphorylation or phosphorylation by other protein kinases (Graves and Roach, 1995; Bischof et al., 2013), interactions with other protein and/or cellular components, and subcellular sequestration (Milne et al., 2001; Xu et al., 2019). In addition, homodimerization excludes ATP from the binding site, thus inhibiting kinase activity (Longenecker et al., 1998; Hirner et al., 2012).

In recent years, several studies have highlighted the importance of CK1δ in neurodegenerative diseases, particularly tauopathies, such as Alzheimer’s disease (AD), Parkinson’s disease (PD), and amyotrophic lateral sclerosis (ALS) (Perez et al., 2011). In addition to having unknown etiology, these illnesses are all characterized by loss of neuronal function, with neurotransmitter deficiency, misfolding, and protein aggregation (Breijyeh and Karaman, 2020). Clinical symptoms are manifested differently, depending on the neuronal area involved (Lee et al., 2001).

AD is a progressive neurodegenerative disorder that mainly involves the neurons of the hippocampus (Selkoe, 2001). On the extracellular side, the main marker of the disease is represented by the accumulation of β-amyloid peptides, produced by β-secretase 1 and γ-secretase enzymes, which lead to neuronal death (J and DJ, 2002). Meanwhile, on the intracellular part, the illness presents lesions related to both cytoplasmic accumulations of vacuoles with abnormal dimensions and dense granular content and the assembly of fibrils and filaments within the neuronal body. These types of lesions are characterized by the accumulation of hyperphosphorylated Tau protein not only in the filaments, but also within the vacuoles (Ghoshal et al., 1999).

The correlation between CK1δ activity and tau protein aggregates in various neurodegenerative diseases has been confirmed by co-immunoprecipitation studies, which highlight that the presence of CK1δ is associated with hyperphosphorylated tau aggregates (Schwab et al., 2000; Li et al., 2004). CK1δ phosphorylates tau protein at the Ser202/Thr205 and Ser369/Ser404 residues *in vitro* (Li et al., 2004; Perez et al., 2011). The phosphorylation sites are the same as those involved in binding with tubulin, highlighting the key role of kinase in the pathogenesis of AD (Li et al., 2004). It is not clear whether the hyperactivity of CK1δ is due to an overtranscription of its gene, altered protein turnover, or both causes, but it has been observed that the concentration of the protein CK1δ in an AD-affected hippocampus is 30 times higher than normal (Ghoshal et al., 1999).

In PD, on the other hand, the pathology is characterized by the accumulation of Lewy bodies, consisting of aggregates of α-synuclein hyperphosphorylated by CK1δ at the level of Ser129 residues (Okochi et al., 2000). This process determines a massive loss of neuronal function at the substantia nigra level (Surmeier, 2018).

CK1δ also plays a key role in ALS, a neurodegenerative disorder in which intracellular inclusions of TDP-43 (TAR DNA-binding protein) are found in the frontotemporal lobe. It was established that TDP-43 can be phosphorylated by CK1δ at 29 different sites (Kametani et al., 2009).

These pathologies are all characterized by the absence of effective pharmacological therapy: in fact, there are no EMA-approved drugs on the market that can solve, and therefore cure, these diseases, but there are only palliative therapies for the temporary improvement of the patient’s quality of life, thus resulting in a high social cost (Dementia, 2021). For these reasons, CK1δ appears as an interesting therapeutical target in the field of neurodegeneration, as witnessed by the increasing interest in the research for inhibitory candidates for this protein during the last 15 years.

Concerning the identification of novel kinase inhibitors, an approach that has proven to be particularly successful is the so-called fragment-based drug discovery (FBDD), as demonstrated by the approval of the BRAF inhibitor vemurafenib (Bollag et al., 2012) (employed in the treatment of metastatic melanoma) and by several other kinase inhibitors which are at various stages of clinical trials (Erlanson et al., 2016; Schoepfer et al., 2018).

This approach revolves around the exploitation of “fragments,” i.e., compounds that respect the “Rule of Three” (molecular weight <300, number of hydrogen bond donor/acceptor ≤ 3, log *P*≤3), as a starting point for the rational development of novel mature, drug-like, active molecules (Hajduk, 2006; Jhoti et al., 2013). The main reason for the success of FBDD is the ability to sample a larger portion of the chemical space compared to the one occupied by drug-like molecules, thus increasing the success rate in finding novel scaffolds for targets of interest (Hall et al., 2014).

This methodology heavily relies on very sensitive biophysical methods, such as X-ray crystallography (XRC), nuclear magnetic resonance (NMR), or surface plasmon resonance (SPR), to perform large screening campaigns on libraries composed of molecules with low molecular weight and high solubility, to find hit compounds (Erlanson et al., 2004; Murray and Rees, 20092009). These hit fragments usually have a low affinity for the target, ranging from low millimolar to high micromolar (hence the need for very sensitive screening techniques), but a higher binding efficiency compared to traditional drug-like molecules, being able to establish high-quality interaction with the target (Schultes et al., 2010). Fragment hits can then be easily combined (either through a linking or a merging process) or chemically modified (growing) to increase their affinity for the target, allowing for the development of potent and selective active compounds (Rees et al., 2004).

Alongside the aforementioned experimental techniques, in the last decade, a prominent role in FBDD campaigns has been played by computer-aided drug discovery (CADD) techniques, such as molecular docking or molecular dynamics (Bissaro et al., 2020). These computational approaches have been routinely and successfully applied for performing large screening on virtual fragment libraries, for the characterization of the fragment interaction mode with the target and to aid the fragment-to-lead optimization in a less time-consuming, more rational, and more efficient way. Some examples of software specifically designed for FBDD are LUDI (Böhm, 1992), HOOK (Eisen et al., 1994), CAVEAT (Lauri and Bartlett, 19941994), and RECORE (Maass et al., 2007). Moreover, commercial drug discovery suites, such as Schrödinger, MOE, and OpenEye, have implemented several tools related to the fragment optimization process.

Among the plethora of software available for FBDD, the open-source software Autogrow, developed by the Durrant lab, is particularly interesting. As thoroughly described in the work of Spiegel and Durrant (2020), the open-source software Autogrow is a Python written code that combines a genetic algorithm with docking calculation based on the Vina (Trott and Olson, 2010) docking software to perform a semi-automatized process for both *de novo* drug design and lead optimization. The latest release of the Autogrow (version 4.0.3, the one used in this work) was developed with the idea of making the codebase modular, thus allowing the third-party implementation of different conversion scripts, molecular docking programs, scoring functions, and reaction libraries, to better suit the need of different research groups.

A recent scientific work published by our laboratory led to the identification of seven novel fragment compounds that bind the hinge region of CK1δ with a low-micromolar IC_50_ (Bolcato et al., 2021). Attracted by the idea of exploiting a semi-automatized computational protocol for the optimization of our newly discovered fragment compounds, we decided to investigate if this protocol would be suitable for our needs. Since it is notorious that molecular docking programs are usually very efficient and optimized with regard to the conformational search, but are usually lacking in the scoring phase (Chen, 2015; Chaput and Mouawad, 2017) [especially for molecules-like fragments that deviate from the drug-like chemical space on which these scoring functions have been trained (Verdonk et al., 2011; de Souza Neto et al., 2020)], we decided to investigate if the implementation of a different scoring protocol based on protein–ligand interaction fingerprint would improve the performance of the Autogrow protocol, concerning the ability of the program to generate compounds similar to known inhibitors based on their interaction scheme and electrostatic and shape similarity.

## 2 Materials and Methods

### 2.1 Hardware Overview

Each general molecular modeling operation has been performed on a Linux Workstation equipped with an 8 core Intel Xeon® CPU E5-1620 CPU. For more intensive calculations, such as the Autogrow runs, a 64 core AMD Opteron™ Processor 6376 CPU cluster was exploited. Both the workstation and the cluster run Ubuntu 16.04 as their operative system.

### 2.2 Structure Preparation

In the case of protein kinase CK1δ, 23 protein–ligand complexes between the protein and small drug-like molecules are available in the Protein Data Bank (Berman et al., 2000) (PDB ID; 3UYT, 3UZP, 4HGT, 4HNF, 4KB8, 4KBA, 4KBC, 4KBK, 4TN6, 4TW9, 4TWC, 5IH5, 5IH6, 5MQV, 5OKT, 5W4W, 6F1W, 6F26, 6GZM, 6HMP, 6HMR, 6RCG, and 6RCH). The crystals with codes 6RU6, 6RU7, and 6RU8 were not considered in this study because they contain the natural substrate adenosine-5′-diphosphate. One of the structures [PDB ID: 4KB8 (Mente et al., 2013)] is composed of two different CK1δ–ligand complexes. For this reason, the system has been separated into two different entries (namely, 4KB8A and 4KB8B). Because of this, the total number of complexes considered in our study is 24.

Each of the mentioned complexes has been downloaded and properly prepared for subsequent computational analysis with the “Structure Preparation” tool implemented in the Molecular Operating Environment (MOE) (Molecular Operating Environment (MOE), 2021) 2019.01 suite. The missing hydrogen atoms were appropriately added with the MOE “Protonate 3D” program (setting the pH for the protonation at a value of 7.4) and were then energetically minimized according to the AMBER10: EHT (Case et al., 2008) force field implemented in MOE. After the preparation phase, the protein–ligand complexes were properly aligned and superposed with the MOE dedicated tool, to make the binding site coordinates coherent among the different crystallographic structures. These complexes were saved and used at a later stage for the generation of the pharmacophore model (see Section 2.4).

Afterward, each ligand was separated from its respective protein. All the small molecules were collected in a database and prepared for docking calculations exploiting several packages from the QUACPAC OpenEye (QUACPAC, 2021) suite. For each molecule, the most probable tautomeric state was selected with the “tautomers” program, the three-dimensional coordinates were rebuilt using the “Omega” tool, the partial charges were attributed with the “MolCharge” program according to the MMFF94 force field, and finally, the dominant protonation state at pH 7.4 was determined by the “FixPka” tool.

### 2.3 Cross-Docking

Each of the aforementioned 24 CK1δ crystallographic ligands, prepared as described in Section 2.2, was docked inside each of the correspondent 24 CK1δ protein structures exploiting two different molecular docking pieces of software, namely GOLD (Jones et al., 2002) (based on a genetic algorithm, developed and distributed with a commercial license from CCDC) and PLANTS (Korb et al., 2006) (an Ant-Colony-Optimization docking algorithm, developed by the University of Tübingen and free for use for academics).

This approach was chosen to follow the principles of “consensus docking” (Houston and Walkinshaw, 2013), which is based on the fact that data obtained by combining results coming from docking programs that operate in an orthogonal way are associated with higher robustness.

For both GOLD and PLANTS, 10 poses per molecule were collected. The default parameters were used for both protocols. Concerning the choice of the scoring function, Chemscore was selected for GOLD, while PLANTS_ChemPLP_ was selected for PLANTS.

A total of 1,152 (24 ligands × 24 proteins × 2 docking protocols) independent docking runs were performed, and the results were then analyzed using an in-house Python script. The script collects the RMSD between each docking pose and the correspondent crystal reference pose, outputting two different plots. The first plot is a heatmap that illustrates the RMSD values for the best docking pose generated for each ligand onto each protein. The second plot is a histogram that re-elaborates the previous results to give a visual representation of the “success rate” of each protein: a successful docking run is obtained when the RMSD between the docking pose and the crystal reference is below the arbitrarily chosen 2 Å threshold value so that the “success rate” is defined by the percentage ratio of the successful docking runs for each protein (i.e., the percentage of docking experiments where the RMSD falls below the threshold value).

### 2.4 Pharmacophore Modeling

Based on previously published works on the same target, we took advantage of the structural information about known inhibitors of CK1δ in the form of crystal structures of their complex with the kinase deposited in the PDB. The same 24 protein–ligand complexes mentioned in Section 2.2 were subjected to the MOE Pharmacophore model tool: shared interaction features (with a 50% threshold value for feature retention) were then used in the generation of the pharmacophore model.

**FIGURE 1:**
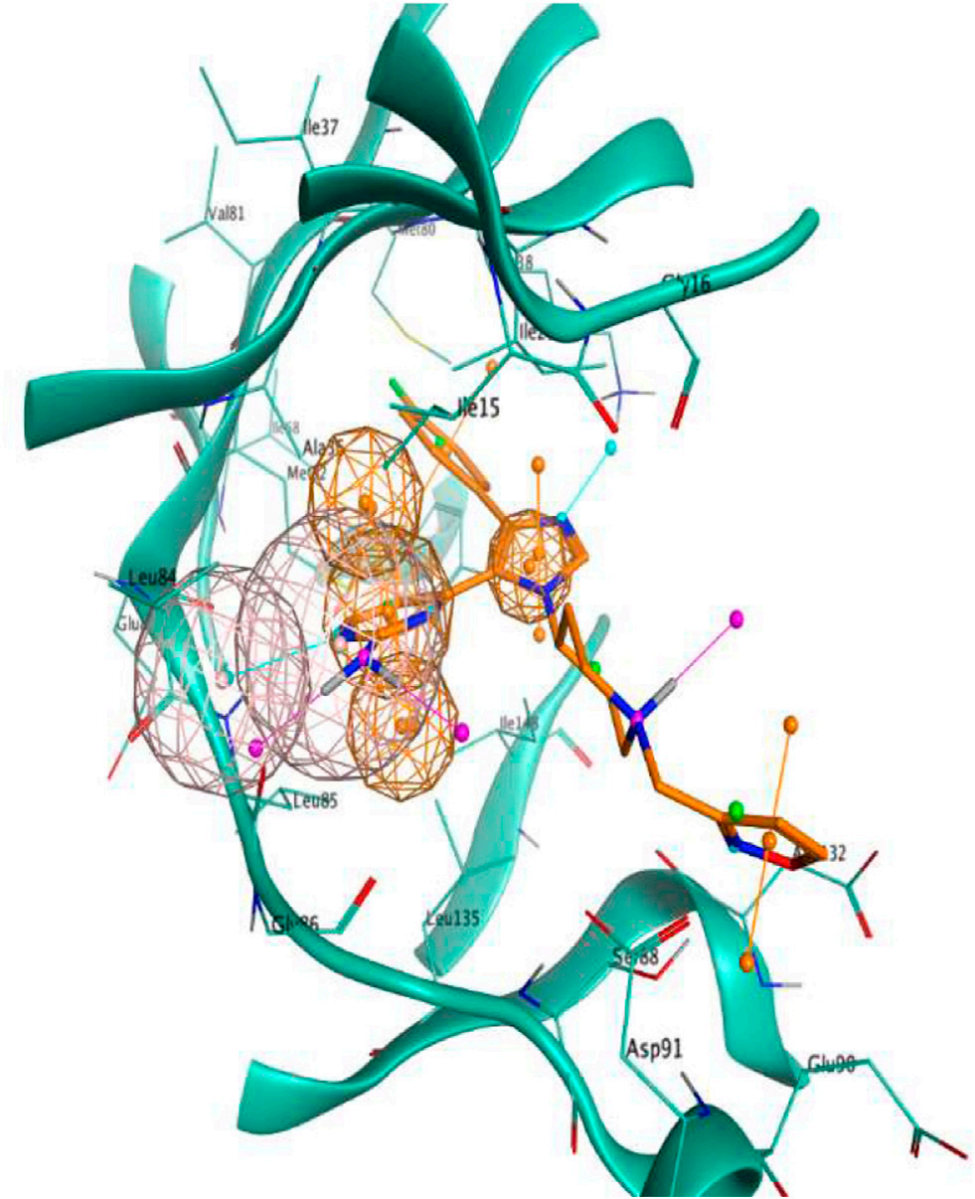
Visual representation of the pharmacophore model used in this scientific work. Features are represented as spheres. Orange spheres indicate an aromatic ring, with an orientation determined by the small orange pin, while the pink spheres indicate a hydrogen bond donor/acceptor. For visual reference, the 4TN6 complex is also reported in this figure, with the protein represented in teal ribbons and the PFO ligand represented as orange sticks.

As can be seen in Figure 1, the final model consisted of four features (represented as spheres in the image), namely a hydrogen bond donor and a hydrogen bond acceptor interacting with Leu85, an aromatic ring in the proximity of the hinge region, and another aromatic ring adjacent to the first one in the inner part of the binding pocket.

### 2.5 Autogrow

Autogrow4 (AutoGrow4, 2020) is a fully open-source code written in Python and developed by the Durrant lab that combines a genetic algorithm with docking calculation based on the Vina (Eberhardt et al., 2021) docking software (version 1.2.0) to perform a semi-automatized process for both *de novo* drug design and lead optimization.

Molecules are submitted to the program in the form of SMILES strings. The genetic algorithm part of the code uses a series of synthetically feasible reactions to perform a defined number of mutation and crossover operations (i.e., growing and merging) on submitted chemical entities, creating a full population (called generation) of molecules to feed to the molecular docking program.

This generation is then docked using the Vina docking software. After the docking stage, the genetic algorithm retrieves the score for each docking pose, which it uses to rank molecules and pick the most fitted members of the generation to promote them to the next generation. This iterative process is repeated for a user-defined number of generations or until an earlier termination criterion is met.

The code is released under the Apache2 license, is freely available at https://durrantlab.pitt.edu/autogrow4/, and works both in Python 2.7 and ≥3.6 environment. A detailed description of how the latest Autogrow release works is provided in the work of Spiegel and Durrant (2020).

Two different versions of the Autogrow code were used in this scientific work. The first one was downloaded from the official repository and used as is, without any modifications to the source code. The second one was the result of an in-house modification of the source code performed to customize the scoring stage of the docking process. The traditional Autogrow protocol uses the Vina standard scoring function (from now on, defined as VINA), which encompasses some elements of knowledge-based potentials and others of a typical empiric scoring function (Eberhardt et al., 2021). Instead, our modified version of the Autogrow code implements an alternative scoring function (from now on, defined as IFP_CS_) based on the similarity between protein–ligand interaction fingerprints.

The crystal complex of a known inhibitor is chosen as reference (in our case, the ligand PFO from complex 4TN6 was chosen) and its binding mode is codified into a bit vector exploiting the InteractionFingerprint function from the fingerprint module of the Open Drug Discovery Toolkit (Wójcikowski et al., 2015) Python Library. This function converts the protein–ligand interaction into a bit array according to the residue of choice and the type of interaction. Each protein residue is represented by eight bits, one for each type of interaction considered (hydrophobic contacts, aromatic face to face, aromatic edge to face, hydrogen bond with protein acting as donor, hydrogen bond with protein acting as acceptor, salt bridge with protein acting as the positively charged member, salt bridge with protein acting as the positively negative member, and ionic bond with a metal ion), so that the final vector will have a size of *r*×8, where *r* stands for the number of protein residues.

During the scoring phase of our custom Autogrow run, each docking pose is also codified into an Interaction Fingerprint vector, the same way as for the crystal reference. Then, the two vectors are transformed from sparse to dense, making use of the appropriate functions from the Numpy Python library, before the comparison between the reference and the query fingerprint is executed using the cosine similarity metrics, exploiting the appropriate function of the Scikit-learn Python library. The resulting score, which ranges from 1 (indicating a complete agreement and coherence between the two binding modes) to 0 (indicating that the two binding modes are not coherent), is then multiplied by −1 to comply with the selection mechanism of Autogrow genetic algorithm, which favors the most negative scores, as is usually the case for most classic scoring functions, like the one used by Vina.

$$IFP_{CS}=\;\frac{A\cdot B}{\left\|A\right\|\left\|B\right\|}*\left(-1\right).$$ (1)

Equation 1 is the mathematic formulation of the IFP_CS_ scoring function. This scoring function is the inverse of the cosine similarity between two vectors, A and B, representing the Interaction Fingerprint for the reference and the query ligand, respectively. Values range from −1 (indicating maximum coherence between the two binding modes) to 0 (indicating the lowest possible correspondence between the two binding modes).

## 3 Results

### 3.1 Cross-Docking

Since 24 different protein–ligand complexes were available for CK1δ (the target for our computational study), but only one at a given time can be used for docking calculations, we had to carefully evaluate the one most suitable for our needs. The choice of the protein structure to use for docking calculation is not trivial, for several reasons. When a ligand gets in contact with a protein, the binding event may cause a change in the structure of the protein itself (Li and Ji, 2019). These modifications are mainly depictable in the binding site and may also be extended to other regions. In a crystallographic complex, this effect is highlightable by differences in the shape of the binding site among the different crystal structures available for a single protein (Csermely et al., 2010).

One of the possible approaches to accomplish this task, the one that we used in our workflow, is known as “cross-docking” (Wierbowski et al., 2020). This technique consists in taking all the protein–ligand complexes available for a target, separating all the ligands from their respective co-crystallized structure, and docking all the different ligands in the binding site of each different protein structure. By analyzing the docking results, it is possible to define the crystallographic protein structure that has the highest tendency to correctly reproduce ligands’ crystallographic conformation.

**FIGURE 2:**
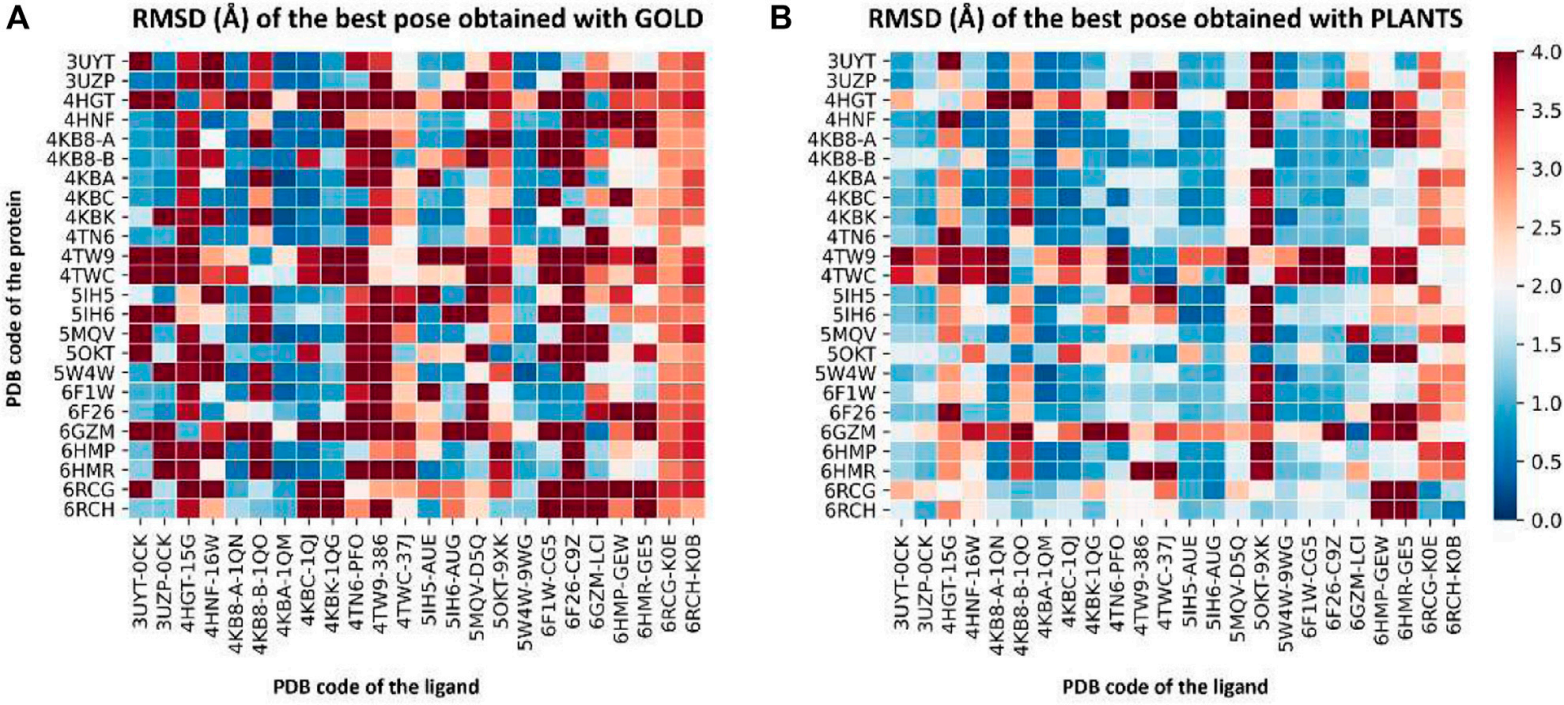
Two heatmaps that summarize the results of the cross-docking experiment performed before the Autogrow runs to select the protein structure to use for subsequent calculations. Panel **(A)** reports the results for the GOLD-Chemscore protocol, while Panel **(B)** encompasses the results of the PLANTS-PLANTS_ChemPLP_ one. On the vertical axis, the PDB code of the protein is reported, while on the horizontal axis the PDB code of the ligand is indicated. The colored squares report the RMSD values for the best docking pose generated by the two docking protocols according to the color bar located on the right side of the image: color ranges from blue (indicating a low RMSD; minimum value is 0 Å, indicating a perfect superposition between the docking pose and the crystal reference) to red (maximum value is 4 Å, indicating a high deviation between the docking pose and the crystal reference).

For these reasons, we performed a cross-docking experiment on our 24 CK1δ complexes to decide which one to pick for subsequent calculation. Each ligand was docked into each protein structure using two different docking protocols, GOLD-Chemscore and PLANTS-PLANTS_ChemPLP_, for a total of 1,152 independent docking runs. For each ligand, the root-mean-square deviation (RMSD) between each docking pose and the crystallographic conformation was calculated. The poses with the lowest RMSD in each docking run were selected and their RMSDs were plotted, obtaining the graphs represented in Figure 2. A detailed description of the methodology used for the cross-docking experiment is provided in Section 2.3.

To visualize the results more clearly, the data from the plots reported in Figure 2 were re-elaborated to obtain a single indicator of the performance of each protein in reproducing the correct binding mode for docked ligands. We opted for calculating the “success rate” for each protein structure: a 2 Å threshold value was chosen to discriminate between successful and unsuccessful docking runs. For each protein, the percentage of successful docking runs (the “success rate”) was calculated accordingly and plotted in a histogram.

**FIGURE 3:**
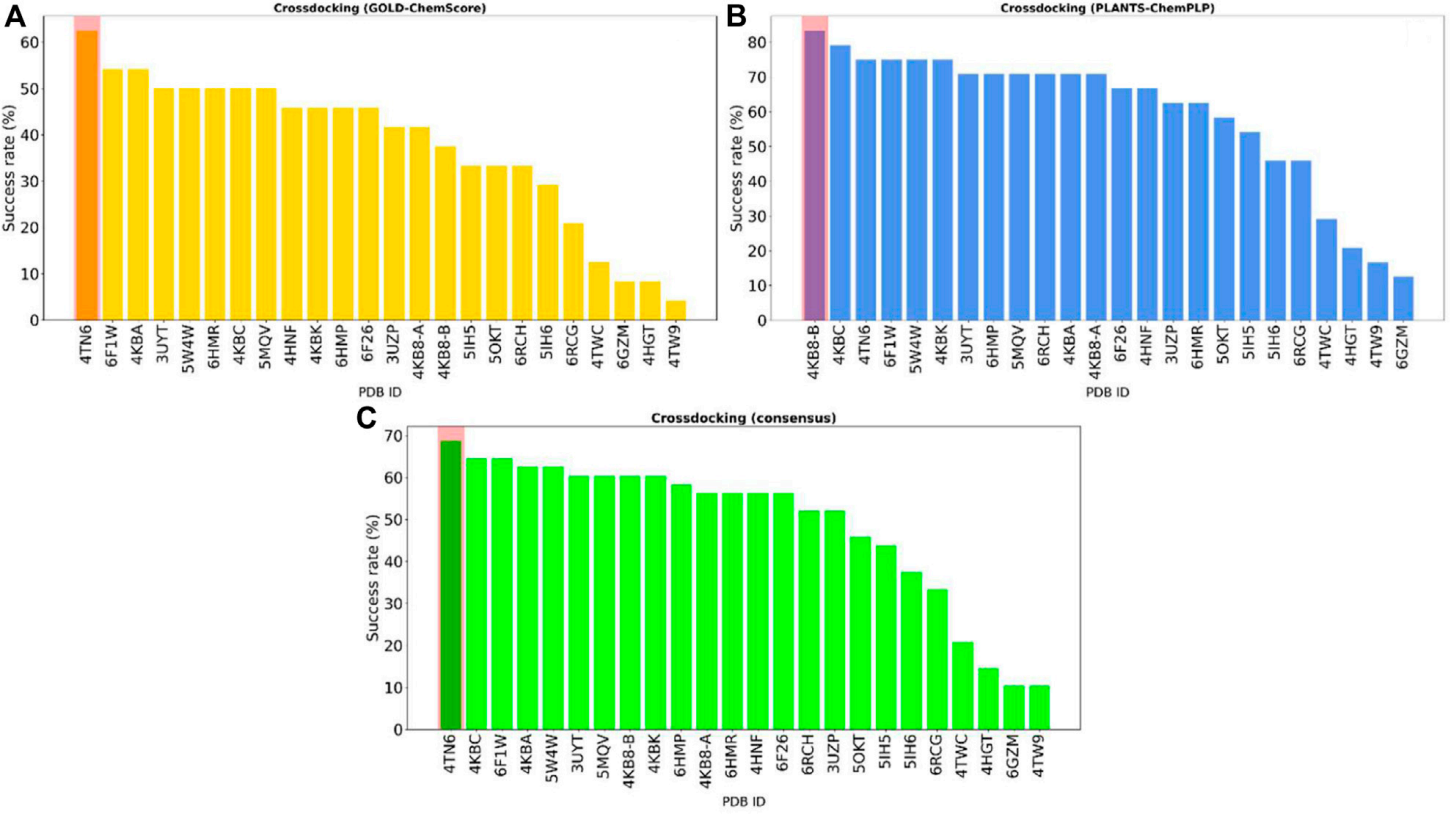
The overall “success rate” in reproducing the correct crystallographic binding mode for each of the 24 CK1δ complexes considered in the study. The “success rate” is defined as the percentage of successful docking runs for each protein in the cross-docking experiment, where a successful docking run is defined as a docking calculation where the RMSD between the best docking pose and the crystal reference falls below an arbitrarily chosen threshold value of 2 Å. Panel **(A)** reports the results for the GOLD-Chemscore protocol. Panel **(B)** reports the results for the PLANTS-PLANTS_ChemPLP_ protocol. Panel **(C)** encompasses the combined “success rate” for each protein, defined as the average between the success rate for each protocol. Protein from the complex 4TN6 was chosen as the most representative CK1δ structure for successive calculations.

Figure 3 encompasses the results of this second analysis, reporting the success rate for both the GOLD-Chemscore and PLANTS-PLANTS_ChemPLP_ protocols. Moreover, since we adopted the principle of “consensus docking,” as mentioned in Section 2.3, we decided to calculate the average success rate between the two docking protocols. As can be seen in Figure 3, the overall “success rate” obtained by the combination of data from the two docking protocols indicates the protein from the complex 4TN6 as the protein that is, on average, more able than the other ones to correctly reproduce the crystallographic binding mode of docked ligands. Although the difference in the success rate between the first and the second protein is low, in the context of several consequential docking runs where thousands of compounds are considered at a given time, even small differences in the percentage success rate could have a big impact on the quality of the run, considering that the prioritization of compounds from one generation to another is based upon their docking-predicted ability to retain the interaction features that characterize the binding mode of known inhibitors. For this reason, we used the protein 4TN6 as a representative CK1δ structure for our subsequent calculations with Autogrow.

### 3.2 Benchmark *De Novo* Run

To assess the performance of our alternative, fingerprint-based, Autogrow protocol (defined as IFP_CS_, while the traditional one is VINA), we first performed a benchmark *de novo* run, using the same conditions as the ones described in the work of Spiegel and Durrant (2020).

A 30-generation run was performed for each protocol, using the “Fragment_MW_100_to_150.smi” library provided in the Autogrow repository and described in the original publication. Configuration files for both *de novo* runs in the JSON format are available in the Supplementary Material, while a detailed description of both Autogrow and our alternative scoring approach is described in Sections 2, 2.5.

**FIGURE 4:**
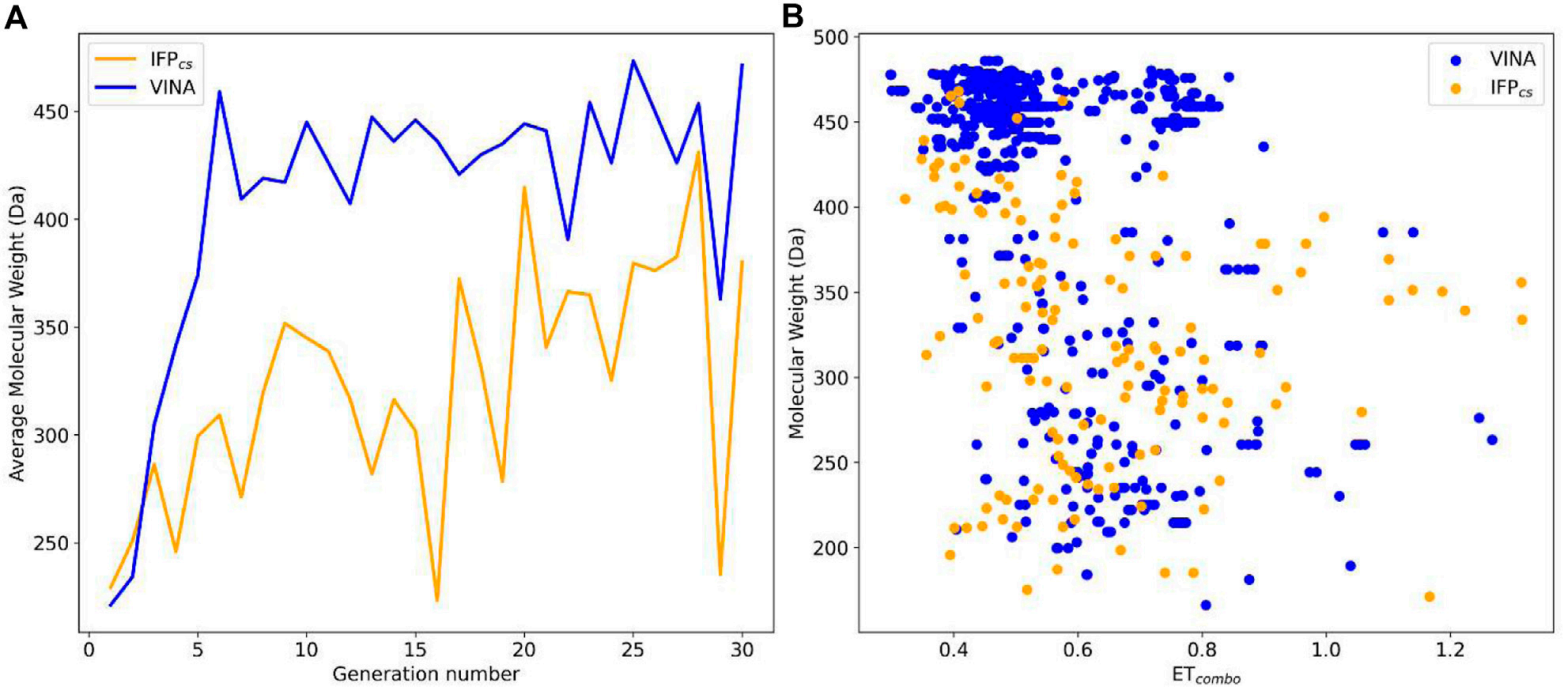
Comparison of the performance of the two Autogrow protocols in the benchmark *de novo* runs regarding their ability to generate compounds that pass the pharmacophore filter. The VINA protocol is reported as a blue line, while the IFP_CS_ one is reported as an orange line. Panel **(A)** depicts, for each protocol, the average molecular weight of compounds within the population that pass the pharmacophore filter on a per-generation basis. The vertical axis reports the molecular weight, while the horizontal axis reports the generation number. Panel **(B)** depicts, for each protocol, the distribution of generated compounds that pass the pharmacophore filter regarding their molecular weight and the similarity of shape and electrostatic properties to crystal inhibitors taken as reference. The vertical axis reports the average molecular weight in Da, while the horizontal axis reports the *ET*_*combo*_ value. Blue dots represent compounds generated by the VINA protocol, while orange dots represent compounds generated by the IFP_CS_ one.

**FIGURE 5:**
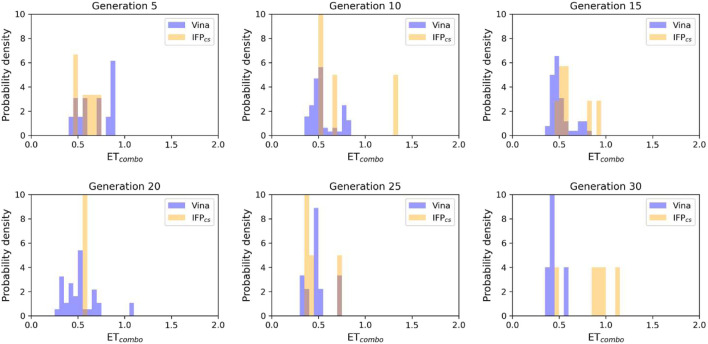
The ability of the two Autogrow protocols in the benchmark *de novo* run to produce compounds that have a high degree of similarity concerning shape and electrostatic properties to the crystallographic ligands, chosen as reference. The probability distribution of the *ET*_*combo*_ score for compounds populating each generation is reported as a histogram, where the vertical axis reports the probability density while the horizontal axis reports the *ET*_*combo*_ value. Two distributions are reported within each plot: the blue bars refer to compounds generated with the VINA protocol, while the orange bars refer to compounds generated with the IFP_CS_ one.

In order to validate the performance of both protocols, we opted for evaluating the quality of the generated compounds by filtering each generation of poses using a pharmacophore model. This filter, which has already been proved to identify true binders in previous related works (Cescon et al., 2020; Bolcato et al., 2021), was used to retain only those poses which complied with known requirements for binding to the CK1δ pocket. This metric was used to determine if there is any advantage in incorporating a knowledge-based element in the generation of novel potential inhibitors of CK1δ, steering the compound selection process toward the ones that assume a pharmacophore-like binding mode. These pharmacophore-like compounds were then characterized by calculating their molecular weight and the similarity of their shape and electrostatic properties to crystal CK1δ inhibitors taken as reference. For this purpose, the EON (Eberhardt et al., 2021) package from the OpenEye suite was used. Each compound passing the pharmacophore filter was compared with each crystallographic ligand, calculating the electrostatic and shape similarity (*ET*_*combo*_). The best value for each ligand was extracted and used for the elaboration of the *a posteriori* analysis, whose results are reported in Figures 4, 5.

As can be seen in Figure 4A, which shows the average molecular weight of compounds that pass the pharmacophore filter for each generation, the VINA protocol rapidly reaches the peak of the average molecular weight (around generation 6), while our IFP_CS_ protocol has a slower but regular growth that reaches values comparable to the VINA protocol from around generation 27 onwards. This difference is probably related to the fact that the VINA scoring function is biased toward the selection of larger compounds, which can make a good number of non-specific interactions with the target, while our IFP_CS_ one is biased toward the selection of compounds that have a similar interaction pattern compared to a reference compound, regardless of their dimensions.

As depicted in Figure 4B, which illustrates the distribution of generated compounds across all generations concerning their molecular weight and their electrostatic and shape similarity with crystal CK1δ inhibitors, this different selection process results in the production of compounds with different properties: the blue dots, which represent the compounds generated by the VINA protocol, are mostly located in the left-upper portion of the graph, indicating that most of the compounds generated by the traditional protocol have a high molecular weight but a low level of similarity with known inhibitors. On the contrary, the upper-right part of the graph (high molecular weight, high electrostatic, and shape similarity) is mostly populated with orange dots, which represent the compounds generated by our IFP_CS_ protocol.

**FIGURE 6:**
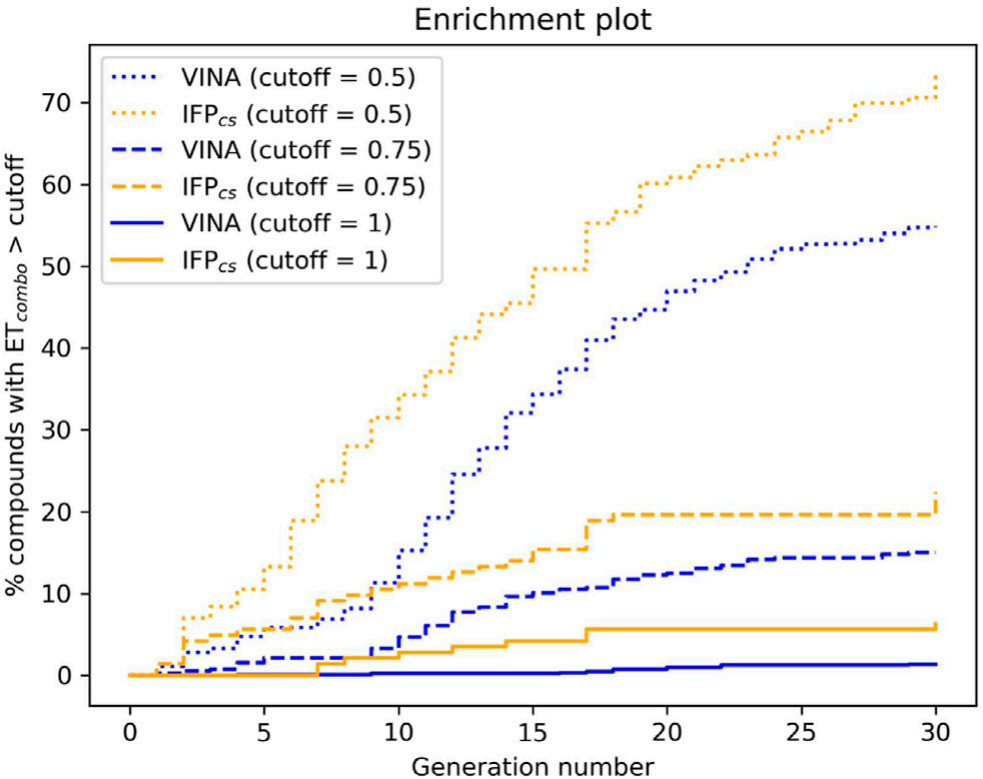
The capability of the two different Autogrow protocols in the benchmark *de novo* run to produce compounds that have a high degree of similarity concerning shape and electrostatic properties to the crystallographic ligands, chosen as reference. For each generation, the percentage of compounds within the total population whose *ET*_*combo*_ exceeds a defined threshold value is reported. Three different cutoff values are reported: 0.50, 0.75, and 1.00, respectively.

**FIGURE 7:**
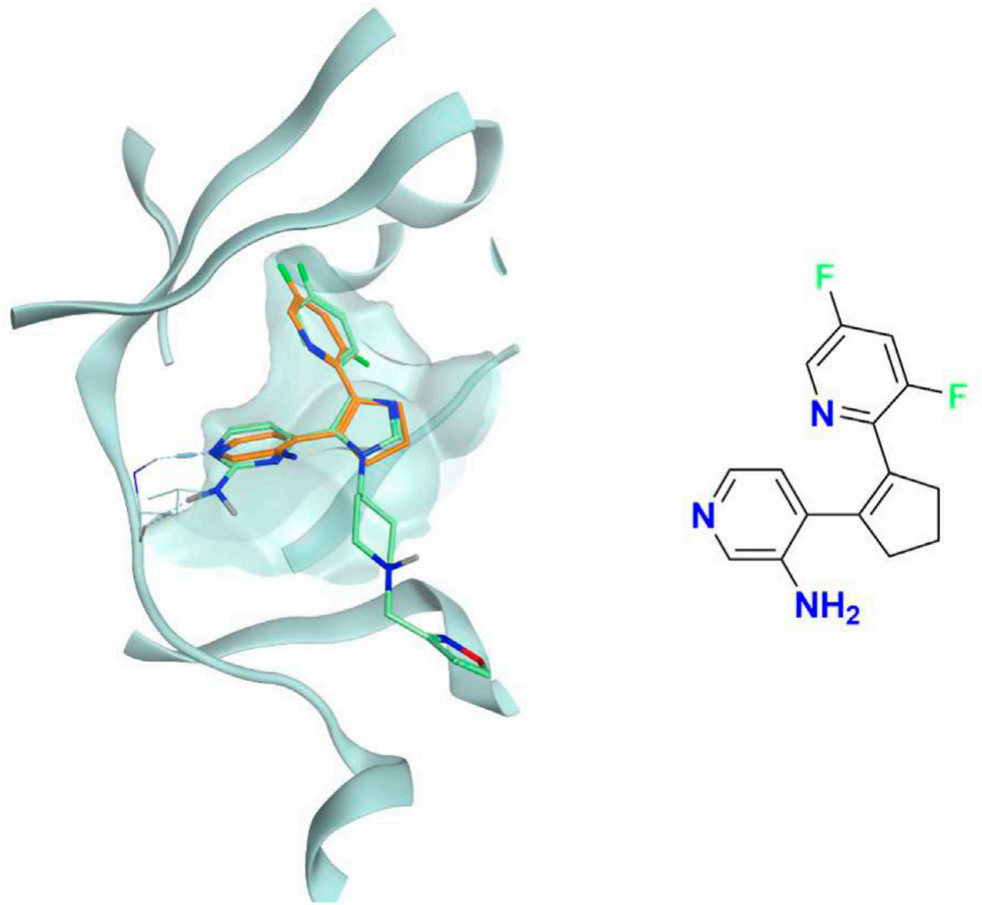
The superposition between the docking-predicted binding mode of a high-scoring compound (MMS1) from the benchmark *de novo* run performed with the IFP_CS_ scoring protocol and the reference crystal binding pose of compound PFO from the structure deposited in the Protein Data Bank with accession code 4TN6. On the left part of the image, the protein kinase CK1δ ATP binding site is reported in teal ribbon, the pose of the compound MMS1 is shown as orange sticks, while the pose of compound PFO is shown as green sticks. On the right part of the image, the chemical structure of the compound MMS1 is reported.

The difference in the selection process is also highlighted in Figure 5, which illustrates the distribution of compounds across a representative subset of generations concerning their electrostatic and shape similarity: the graph clearly shows how the VINA protocol does not improve the similarity of generated compounds while increasing the number of generations. On the contrary, the orange population (which represents the compounds generated by the IFP_CS_ protocol) gradually shifts toward the right part of the plot passing from earlier to later stage generations, indicating that the compounds passing the pharmacophore filter increase their electrostatic and shape similarity passing from one generation to another. Another comparison of the performances of the two protocol is given in Figure 6, which reports the progressive enrichment in compounds with a high degree of similarity to reference inhibitors within the total population. An example of a high-scoring compound generated by our IFP_CS_ protocol is reported in Figure 7, where its chemical structure and the comparison between its docking-predicted binding mode and the crystal pose of the PFO ligand from reference crystal complex 4TN6 is shown.

### 3.3 Benchmark Lead-Optimization Run

To further evaluate the validity of our custom scoring protocol, we also performed a benchmark lead-optimization run, using once again the same conditions as the ones reported in the work of Spiegel and Durrant (2020).

A 5-generation run was performed for each protocol, using a library composed of the 24 crystallographic ligands mentioned in the previous sections and another 316 fragments obtained from the fragmentation of crystallographic ligands exploiting the “fragmenter_of_smi_mol.py” Python script provided by the Autogrow developers, using the BRICS fragmentation rule, for a total of 340 compounds fed to the algorithm. In this case, configuration files for both benchmark runs in the JSON format are available in the Supplementary Material.

To assess the performance of both protocols, we applied the same criteria described previously for the *de novo* runs, focusing once again on compounds passing the pharmacophore filter described in Section 2.4 and characterizing them about their molecular weight and electrostatic and shape similarity compared to crystal CK1δ inhibitors.

**FIGURE 8:**
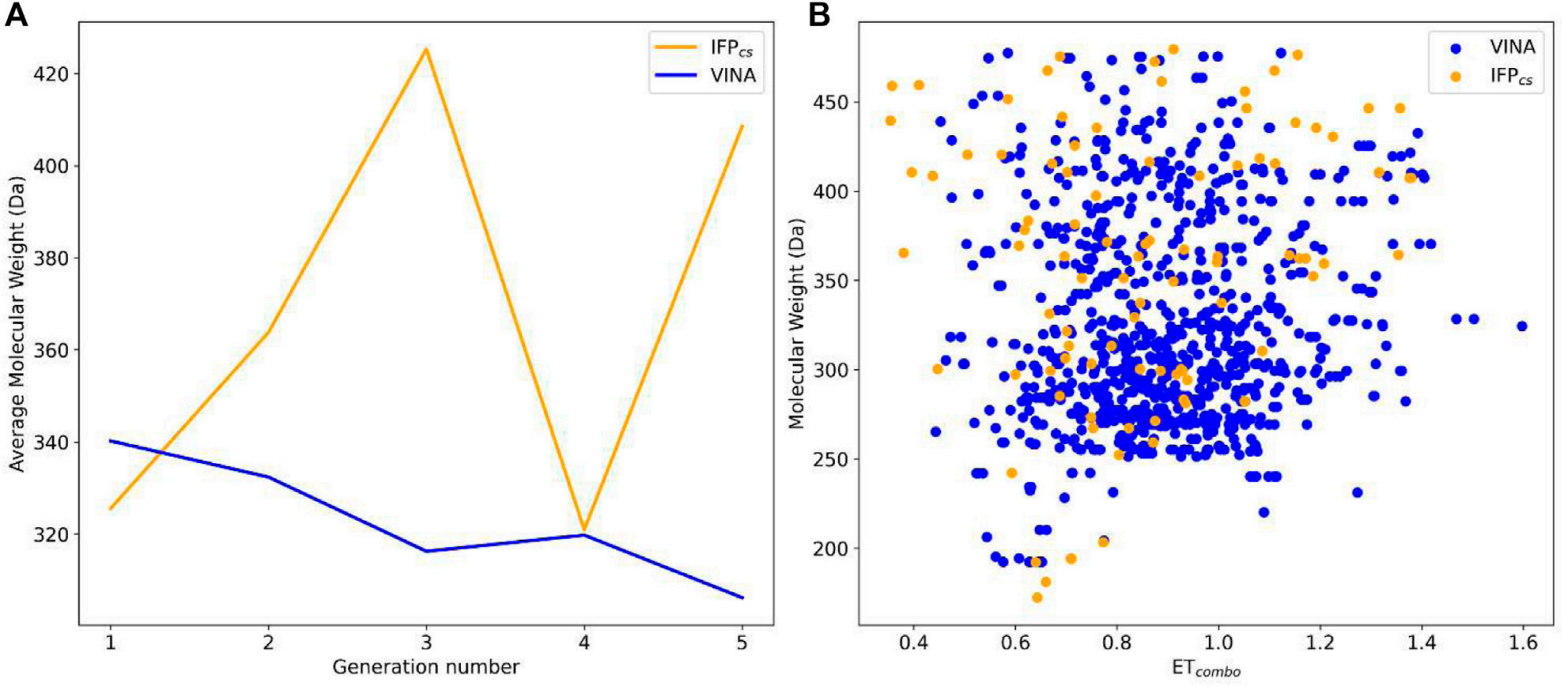
Comparison of the performance of the two Autogrow protocols in the benchmark lead-optimization runs regarding their ability to generate compounds that pass the pharmacophore filter. The VINA protocol is reported as a blue line, while the IFP_CS_ one is reported as an orange line. Panel **(A)** depicts, for each protocol, the average molecular weight of compounds within the population that pass the pharmacophore filter on a per-generation basis. The vertical axis reports the molecular weight, while the horizontal axis reports the generation number. Panel **(B)** depicts, for each protocol, the distribution of generated compounds that pass the pharmacophore filter regarding their molecular weight and the similarity of shape and electrostatic properties to crystal inhibitors taken as reference. The vertical axis reports the average molecular weight in Da, while the horizontal axis reports the *ET*_*combo*_ value. The blue dots represent compounds generated by the VINA protocol, while the orange dots represent compounds generated by the IFP_CS_ one.

**FIGURE 9:**
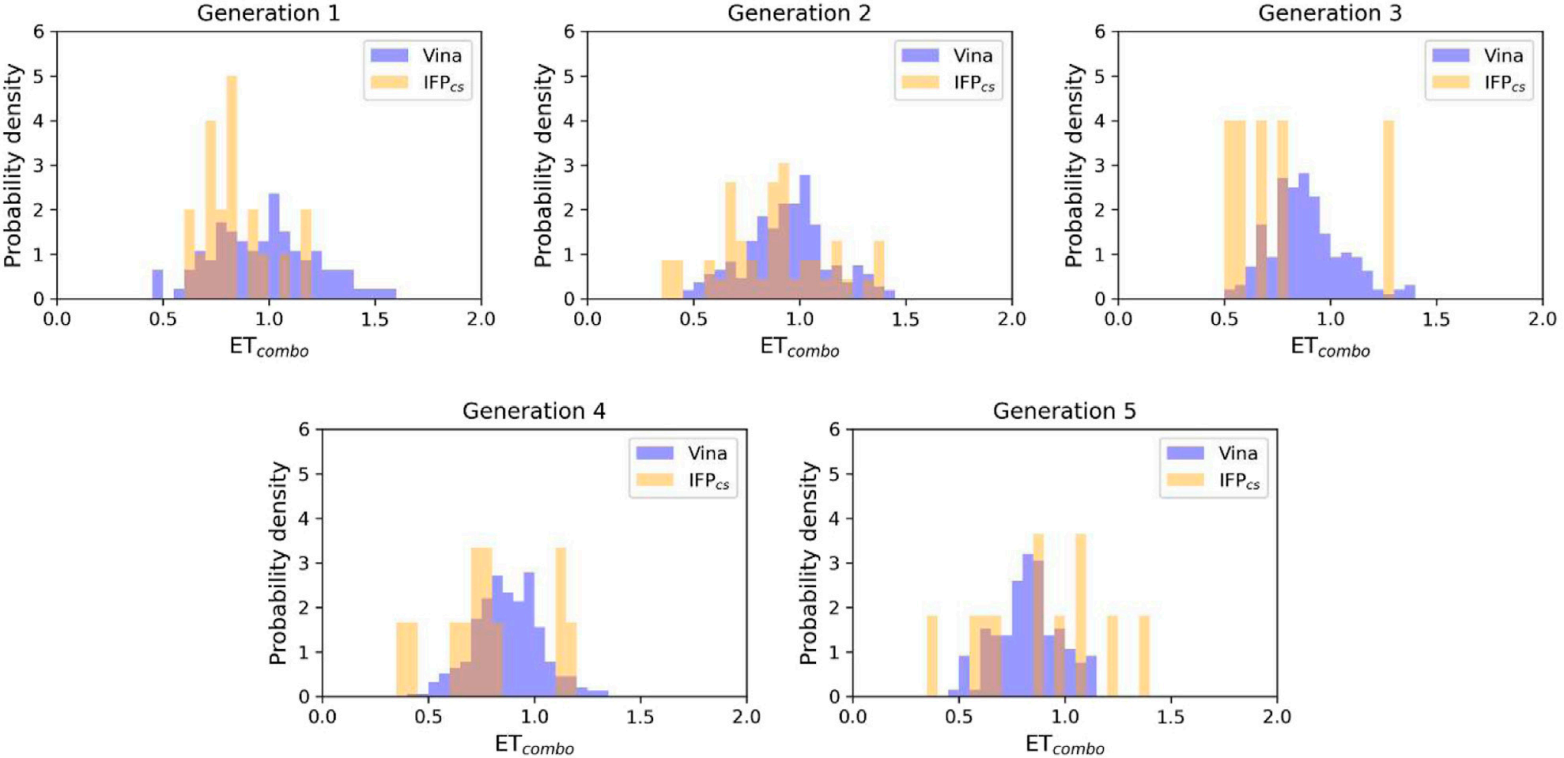
The ability of the two Autogrow protocols in the benchmark lead-optimization run to produce compounds that have a high degree of similarity with regard to shape and electrostatic properties to the crystallographic ligands, chosen as reference. The probability distribution of the *ET*_*combo*_ score for compounds populating each generation is reported as a histogram, where the vertical axis reports the probability density while the horizontal axis reports the *ET*_*combo*_ value. Two distributions are reported within each plot: the blue bars refer to compounds generated with the VINA protocol, while the orange bars refer to compounds generated with the IFP_CS_ one.

**FIGURE 10:**
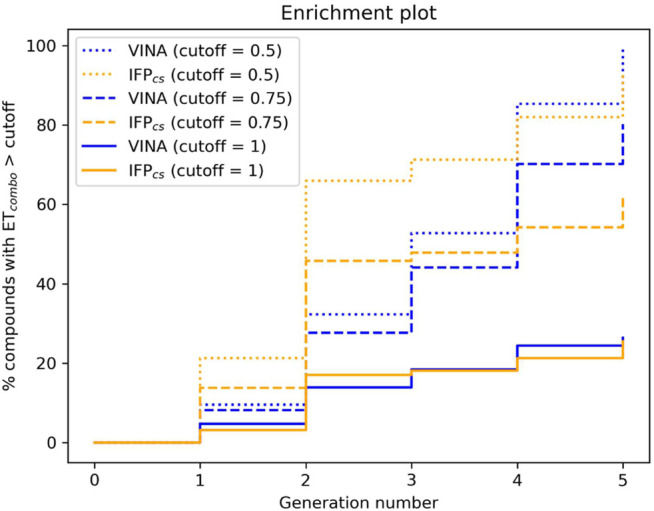
The capability of the two different Autogrow protocols in the benchmark lead-optimization run to produce compounds that have a high degree of similarity concerning shape and electrostatic properties to the crystallographic ligands, chosen as reference. For each generation, the percentage of compounds within the total population whose *ET*_*combo*_ exceeds a defined threshold value is reported. Three different cutoff values are reported: 0.50, 0.75, and 1.00, respectively.

Figure 8B illustrates the distribution of compounds across all five generations regarding their ET_combo_ and their molecular weight: as can be seen, there is little to no difference between the two protocols, with the two populations being practically superimposable. However, contrary to what might be suggested by this plot, there is a significant difference in the performances of the two protocols, which is highlighted in Figures 8A, 9, 10.

**FIGURE 11:**
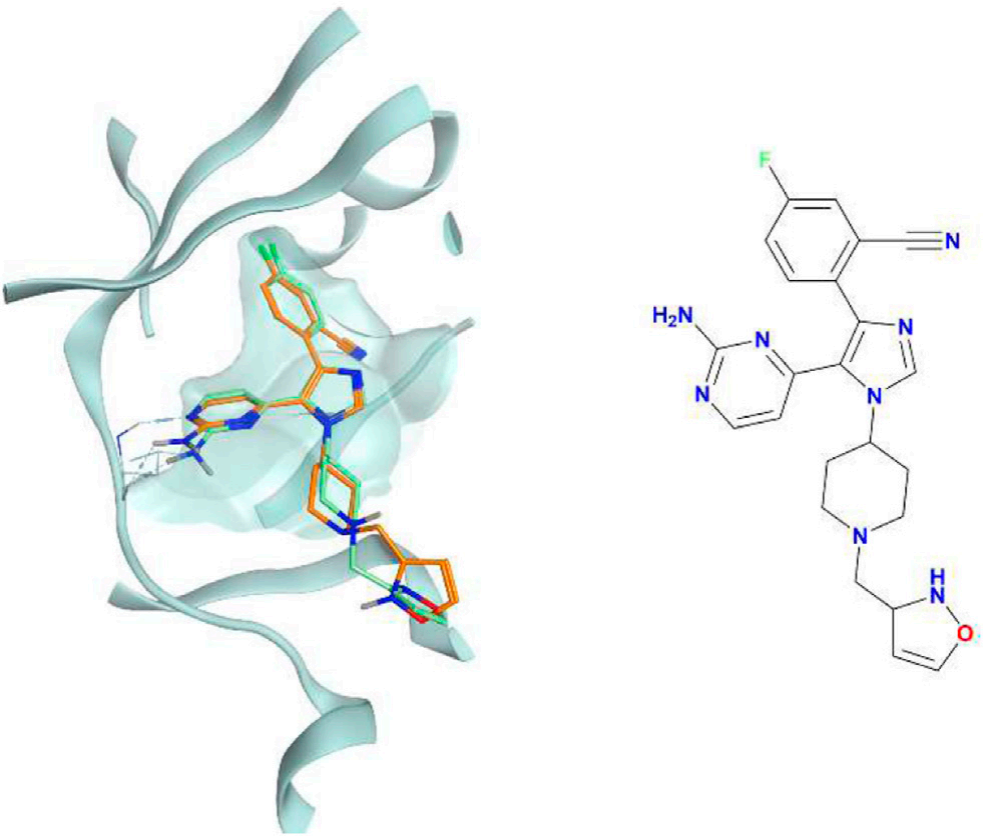
The superposition between the docking-predicted binding mode of a high-scoring compound (MMS2) from the benchmark lead-optimization run performed with the IFP_CS_ scoring protocol and the reference crystal binding pose of compound PFO from the structure deposited in the Protein Data Bank with accession code 4TN6. On the left part of the image, the protein kinase CK1δ ATP binding site is reported in teal ribbon, the pose of the compound MMS2 is shown as orange sticks, while the pose of compound PFO is shown as green sticks. On the right part of the image, the chemical structure of the compound MMS2 is reported.

As can be noticed in Figure 8A, the average molecular weight of compounds passing the pharmacophore filter grows by about 90 Da, passing from the first to the last generation in the case of our IFP_CS_ protocol. On the contrary, the average molecular weight of pharmacophore-like compounds generated by the traditional VINA protocol does not increase with the number of generations, but slightly decreases over time, falling even below the average molecular weight of the first generation derived from the IFP_CS_ protocol. Furthermore, Figure 9 illustrates how, as previously seen in the benchmark *de novo* run, the similarity of compounds passing the pharmacophore filter increases over time when the IFP_CS_ scoring protocol is adopted, while it slightly decreases and does not improve over time in the case of the traditional VINA scoring protocol. Particularly, this trend is also confirmed by Figure 10, which shows how the IFP_CS_ protocol can produce a quicker enrichment of the population in high-similarity compounds compared to the traditional VINA one. As for the previous case, an example of a high-scoring compound generated in the last and final generation of the IFP_CS_ run is reported in Figure 11.

### 3.4 Prospective *De Novo* Run

Encouraged by the results of our benchmark runs, we decided to perform a prospective run with the IFP_CS_ protocol, applying the same operating conditions as before. This time, the starting library was modified to add to the compounds used for the benchmark runs seven fragment ATP-competitive CK1δ inhibitors identified during a previous virtual screening campaign from our laboratory (Bolcato et al., 2021). The idea behind this run was to evaluate the ability of our IFP_CS_ scoring protocol to generate interesting novel potential CK1δ inhibitors derived from in-house, readily available compounds.

**FIGURE 12:**
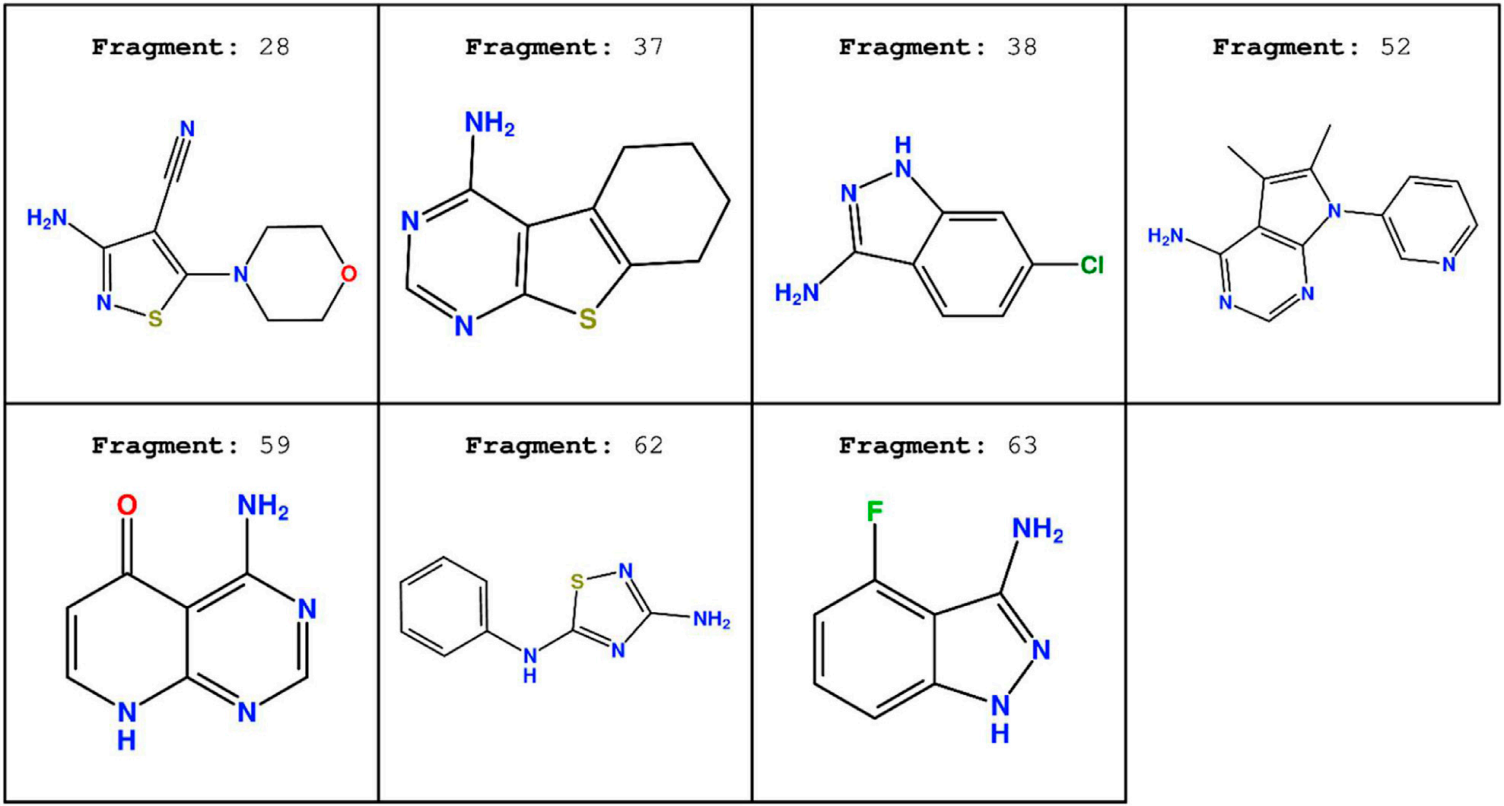
Chemical structure of the seven fragment CK1δ inhibitors derived from the work of Bolcato et al. (2021).

The chemical structure of the seven fragments used in this run is reported in Figure 12.

**FIGURE 13:**
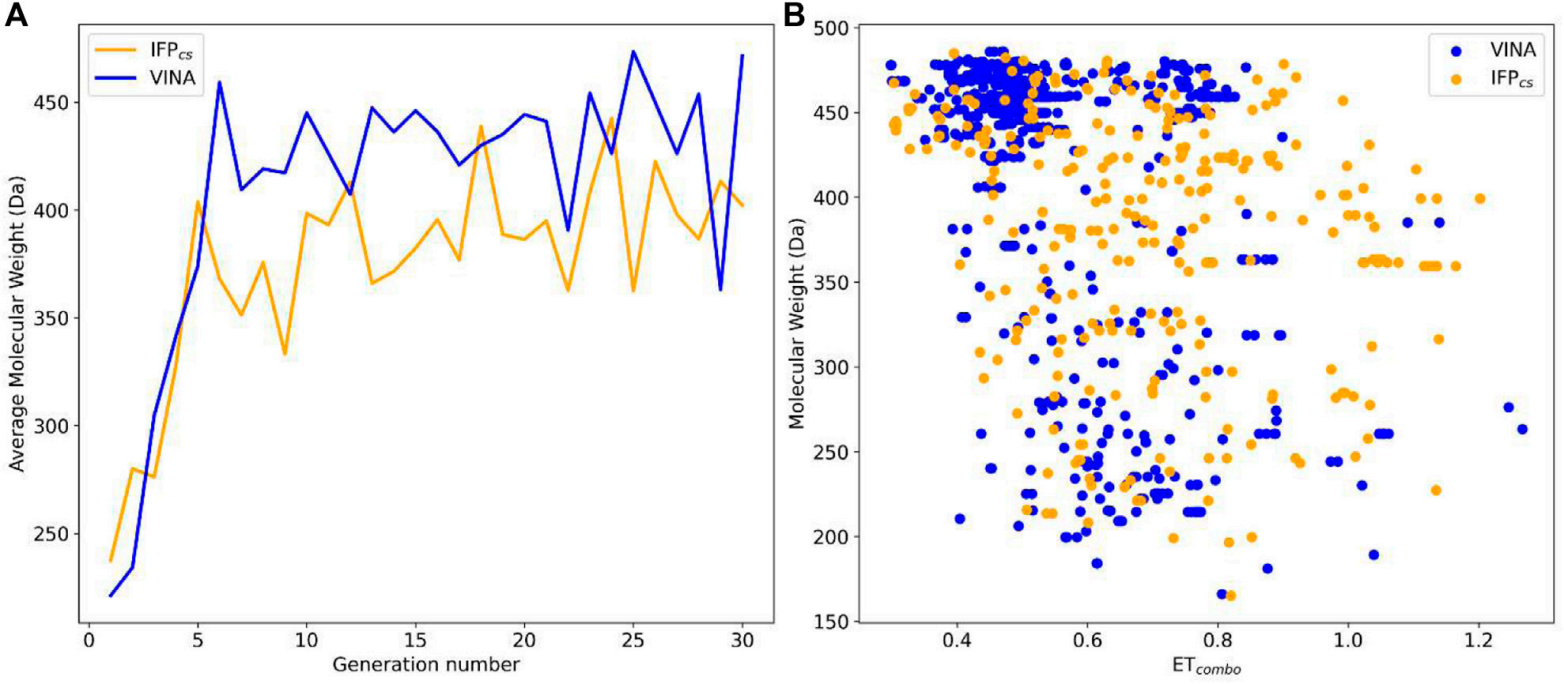
Performance of the two Autogrow protocols in the prospective *de novo* runs regarding their ability to generate compounds that pass the pharmacophore filter. The VINA protocol is reported as a blue line, while the IFP_CS_ one is reported as an orange line. Panel **(A)** depicts, for each protocol, the average molecular weight of compounds within the population that pass the pharmacophore filter on a per-generation basis. The vertical axis reports the molecular weight, while the horizontal axis reports the generation number. Panel **(B)** depicts, for each protocol, the distribution of generated compounds that pass the pharmacophore filter regarding their molecular weight and the similarity of shape and electrostatic properties to crystal inhibitors taken as reference. The vertical axis reports the average molecular weight in Da, while the horizontal axis reports the *ET*_*combo*_ value. The blue dots represent compounds generated by the VINA protocol, while the orange dots represent compounds generated by the IFP_CS_ one.

**FIGURE 14:**
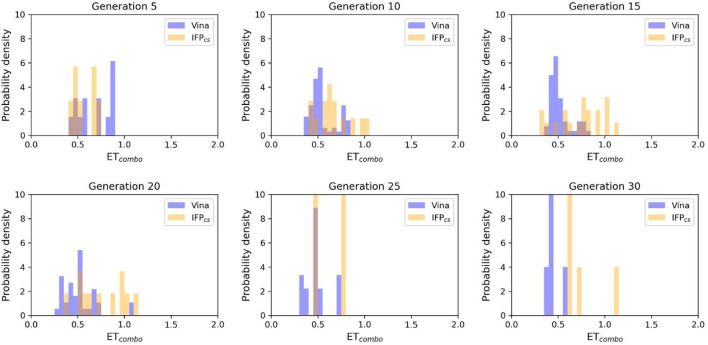
The ability of the two Autogrow protocols in the prospective *de novo* run to produce compounds that have a high degree of similarity with regard to shape and electrostatic properties to the crystallographic ligands, chosen as reference. The probability distribution of the *ET*_*combo*_ score for compounds populating each generation is reported as a histogram, where the vertical axis reports the probability density while the horizontal axis reports the *ET*_*combo*_ value. Two distributions are reported within each plot: the blue bars refer to compounds generated with the VINA protocol, while the orange bars refer to compounds generated with the IFP_CS_ one.

**FIGURE 15:**
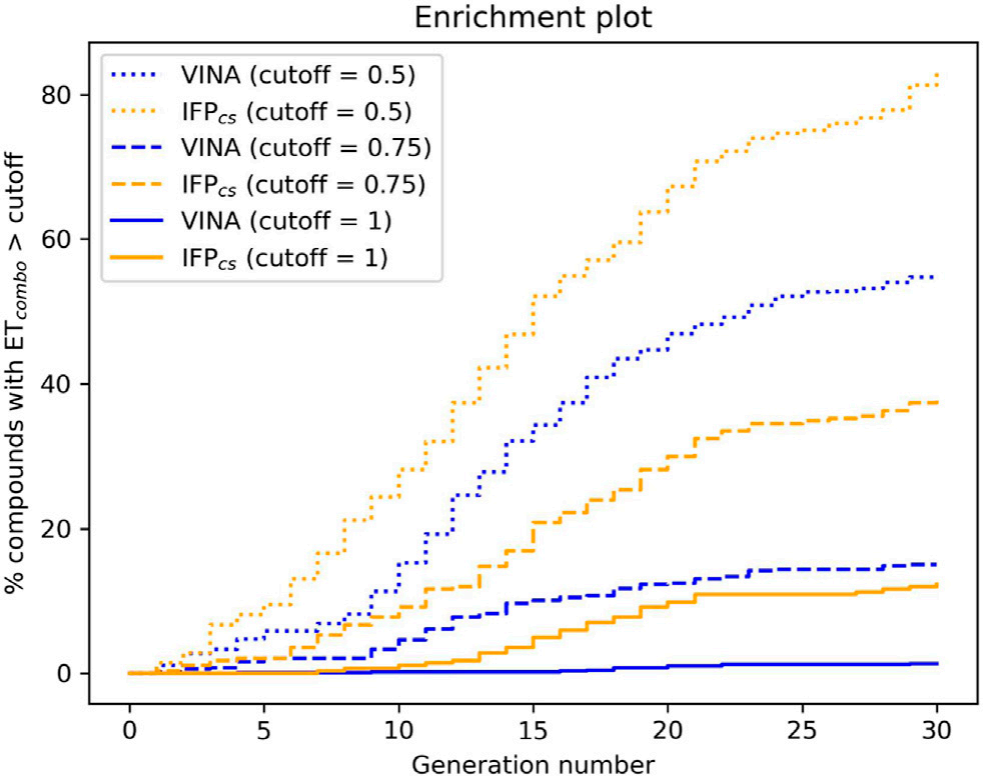
The capability of the two different Autogrow protocols in the prospective *de novo* run to produce compounds that have a high degree of similarity concerning shape and electrostatic properties to the crystallographic ligands, chosen as reference. For each generation, the percentage of compounds within the total population whose *ET*_*combo*_ exceeds a defined threshold value is reported. Three different cutoff values are reported: 0.50, 0.75, and 1.00, respectively.

**FIGURE 16:**
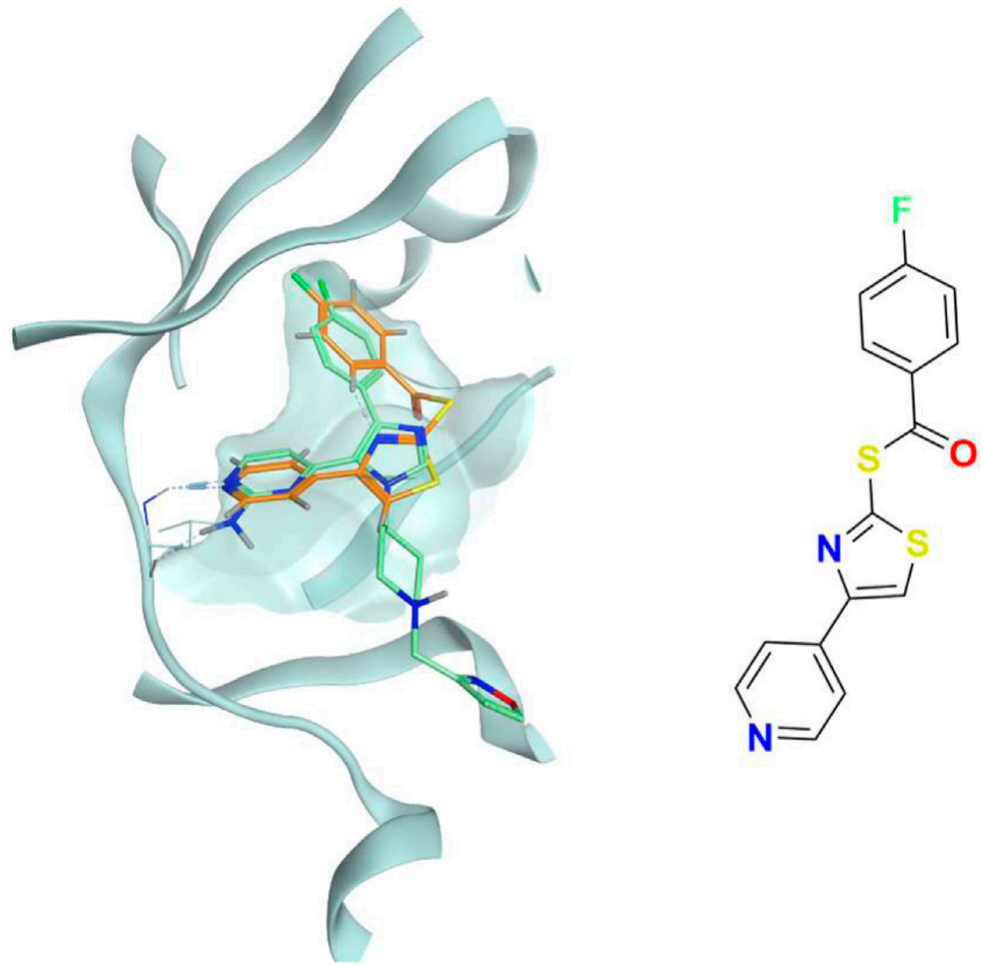
The superposition between the docking-predicted binding mode of a high-scoring compound (MMS3) from the benchmark *de novo* run performed with the IFP_CS_ scoring protocol and the reference crystal binding pose of compound PFO from the structure deposited in the Protein Data Bank with accession code 4TN6. On the left part of the image, the protein kinase CK1δ ATP binding site is reported in teal ribbon, the pose of the compound MMS3 is shown as orange sticks, while the pose of compound PFO is shown in green sticks. On the right part of the image, the chemical structure of the compound MMS3 is reported.

To verify the quality of this run, we performed the same analysis as for the benchmark runs. The results of this analysis are summarized in Figures 13–15, respectively. As remarked in Figure 14, the same trend seen in the benchmark *de novo* run can also be observed in the case of this prospective run: while the VINA protocol is not able to increase the shape and electrostatic similarity to known inhibitors over time, the IFP_CS_ protocol can produce a shift of the orange population toward higher ET_combo_ values. As illustrated by Figure 13, which reports a comparison between the benchmark *de novo* run performed with the VINA protocol and the prospective *de novo* run carried out with the IFP_CS_ protocol, the trend in both the distribution of compounds regarding their molecular weight and ET_combo_ and the growth of molecular weight over time are similar to the benchmark *de novo* run. Figure 13B clearly shows how the upper-right portion of the graph, which hosts compounds with both high molecular weight and ET_combo_ values, is populated exclusively by orange dots, which represent compounds generated with the IFP_CS_ scoring protocol. Interestingly, Figure 14A highlights how there is much less difference in the growth rate of molecular weight between the IFP_CS_ run (which is contaminated by the presence of our seven CK1δ-inhibiting fragments) and the benchmark VINA run, suggesting that performances of the IFP_CS_ could improve if some high-quality pharmacophore-like fragments are included in the starting library. However, despite the quicker growth of molecular weight, the quality of generated compounds follows the same trend seen in the benchmark *de novo* run, as reported in Figure 15. As for the previous cases, an example of a high-scoring compound is reported in Figure 16.

## 4 Discussion

The open-source software Autogrow4 (AutoGrow4, 2020) is an interesting piece of code that utilizes a combination between a genetic algorithm and the Vina (Trott and Olson, 2010) molecular docking software to semi-automatize the processes of fragment growing and lead optimization. Thanks to the modular nature of the codebase, we implemented an alternative scoring protocol (IFP_CS_) based on the similarity of protein–ligand interaction fingerprint between a crystal reference and query compounds, exploiting the appropriate function from the open-source library Open Drug Discovery Toolkit (Wójcikowski et al., 2015), and we compared its performances with the traditional Autogrow scoring protocol (VINA), which is based on the Autodock Vina scoring function.

The VINA protocol uses a scoring function that incorporates some elements of knowledge-based potentials and some others of empirical scoring functions. As is the case for many scoring functions, the score is biased toward higher molecular weight compounds, which can establish a higher number of non-specific interactions with the target (Chen, 2015). For this reason, usually, molecular docking programs are efficient in sampling the conformational space available for the ligand within the binding site but are weaker in prioritizing the right binding mode among a set of reasonable hypotheses generated by the search algorithm (Chaput and Mouawad, 2017). This is especially true in the case of fragments, which deviate from the drug-like properties of compounds upon which traditional scoring functions have been trained (Verdonk et al., 2011).

As thoroughly discussed in the work of Bolcato et al. (Bolcato et al., 2021), one possible solution to the scoring problem is to apply a pharmacophore filter to poses generated by the molecular docking program. When several structural pieces of information are available in the form of protein–ligand crystal complexes for a certain target (as is the case for protein kinase CK1δ, the case study for this work), a good solution to reduce the false positive rate of molecular docking programs is to build a pharmacophore model that encompasses the most prominent interaction features that are required to bind ligands to the target active site (Peach and Nicklaus, 2009). In the case of a program like Autogrow, where the selection mechanism that determines which compounds to promote to the next generation is based on the docking score, we thought it would be interesting to incorporate a knowledge-based element in the pose selection mechanism in the form of a comparison between the interaction fingerprint of query compounds and known inhibitors, to bias the selection mechanism toward molecules that respect the required features to bind to the target.

To validate our IFP_CS_ scoring protocol, we performed both a *de novo* and a lead-optimization benchmark run, using the same operative conditions described in the original work of Spiegel and Durrant (2020) but on a different target. The protein target of choice was the protein kinase CK1δ, a pharmaceutically relevant target in the field of neurodegenerative diseases for which several crystal complexes with inhibitors are available in the PDB. The benchmark *de novo* run was performed on a library composed of 6,103 fragment compounds whose molecular weight falls between 100 and 150 Da, while the benchmark lead-optimization run was carried out on a library composed of 24 crystallographic ligands of the protein kinase CK1δ and 316 fragments derived from the fragmentation of crystallographic ligands using the BRICS rule. To compare the capabilities of the two protocols, we filtered each generation of compounds with the same pharmacophore filter already utilized in previous scientific works on the target (Cescon et al., 2020; Bolcato et al., 2021). We then proceeded to evaluate the quality of compounds that pass the pharmacophore filter, considering both the size and the similarity of shape and electrostatic properties of query compounds compared to the crystallographic ligands taken as reference.

As illustrated by the results of our analysis (Sections 3.3, 3.4, respectively), there is a substantial difference in the performances of the two protocols: while both protocols can generate a certain amount of compounds that pass the pharmacophore filter (therefore possessing the right structural features that are required for the interaction with the target), in both scenarios the IFP_CS_ scoring function outperforms the traditional VINA one regarding the ability to select and prioritize pharmacophore-like compounds that have a similar shape and electrostatic properties compared to known inhibitors of the protein kinase CK1δ. This is particularly evident in the lead-optimization scenario, where within each generation passage, the average molecular weight of compounds that pass the pharmacophore filter steadily increases, passing from the typical MW of a fragment-like compound to the MW of a grown, mature, lead candidate, while the contrary happens in the case of the VINA protocol, with the average MW of the compounds that pass the pharmacophore filter steadily decreasing, falling even below the value of the first generation from the IFP_CS_ protocol. Moreover, when poses from each generation are compared with the ones of crystallographic ligands concerning the shape and electrostatic similarity, a similar trend can be noticed. While the VINA protocol can select high-quality compounds in the first generation, compared to the IFP_CS_ one, at later stages during the run a progressive reduction in the similarity between the query and reference compounds can be noticed, contrary to what happens when the IFP_CS_ scoring protocol is utilized. This can be explained considering the different nature of the two scoring functions: the VINA protocol is biased toward bigger, therefore higher scoring, compounds, while the IFP_CS_ protocol favors compounds that respect the interaction pattern of the reference crystallographic ligand, regardless of their size. For this reason, the IFP_CS_ protocol tends to favor smaller compounds in the first generations, as long as they are complying with the constraint imposed by the reference interaction fingerprint, increasing the possibility to maintain in the population high-quality fragment to be optimized via the mutation and crossover operation of the genetic algorithm. On the contrary, the VINA protocol selects high MW compounds in the first generation that have little to no space for meaningful chemical modifications, giving low priority to those smaller compounds that will have a lower number of interactions with the target, thus resulting in lower docking score. Overall, our IFP_CS_ protocol seems preferable in those cases where structural data are available in the form of protein–ligand complex structure, as is the case for a good number of targets nowadays, while the traditional protocol seems a valid choice in those cases where such structural information is missing.

A recent virtual screening campaign performed in our laboratory led to the identification of seven novel fragment compounds that are ATP-competitive CK1δ inhibitors (Bolcato et al., 2021). Curious to see if our protocol would have been able to produce novel potential CK1δ that incorporates structural features of our seven fragments, we performed a second *de novo* run, using the same conditions as for the benchmark one, except for the introduction in the starting library of those seven fragment compounds. The same analysis performed on the benchmark runs showed that the performance of our IFP_CS_ scoring protocol is even better when the Autogrow protocol is seeded with high-quality fragments that have the right structural feature to interact with the target. Usually, in a typical FBDD campaign, the identification of fragment binders either through virtual or experimental screening leads to the discovery of several potential starting points for the hit-to-lead fragment optimization phase. Our preliminary study showed that it is possible to obtain meaningful results even in those cases where the starting library is populated by fragments that are randomly selected and not specifically tuned for the target of choice, but it certainly benefits from the contamination of the starting library with fragments that are known binders, indicating that the application of the IFP_CS_ protocol could lead to some interesting results in those cases where the known binders constitute a bigger fraction of the starting library. Concerning this, this approach could be utilized to evaluate the competitiveness of newly found scaffolds with the already existing ones, based on the simplicity to derive those scaffolds with common and feasible chemical reactions, therefore producing a good number of derivatives with increased affinity for the target.

## 5 Conclusion

In the present work, we presented and benchmarked a custom version of the open-source Autogrow4 which implements an alternative scoring protocol based on the similarity between protein–ligand interaction fingerprint of query compounds compared to a crystal reference. To demonstrate the applicability of our protocol in a pharmaceutically relevant scenario, we tested its capability to generate compounds that have similar binding and structural features to known inhibitors of the protein kinase CK1δ, a protein that is involved in several neurodegenerative diseases, such as AD, PD, and ALS.

A benchmark *de novo* run and a lead-optimization one were both carried out to compare the performance of our IFP_CS_ scoring protocol against the traditional one implemented in the original version of the Autogrow code, using the same conditions as the one reported in the original publication by Spiegel et al. Compared to the traditional Autogrow protocol, which uses the default scoring function of the Vina docking software, our IFP_CS_ protocol was able to generate, on average, compounds that were bigger and more similar to crystallographic ligands from the point of view of the shape and electrostatic properties, while retaining the key protein–ligand interaction features required for the inhibition of CK1δ.

The custom Autogrow version used in this work, which implements our alternative IFP_CS_ scoring protocol, along with the JSON configuration files used for each run and a YAML file to reconstitute the Python environment to run the custom version of the code, is available in the Supplementary Material.

## Data Availability

Publicly available datasets were analyzed in this study. These data can be found here: https://www.rcsb.org/.
